# Proteomic Approaches to Biomarker Discovery in Cutaneous T-Cell Lymphoma

**DOI:** 10.1155/2016/9602472

**Published:** 2016-10-16

**Authors:** Alexandra Ion, Iris Maria Popa, Laura Maria Lucia Papagheorghe, Cristina Lisievici, Mihai Lupu, Vlad Voiculescu, Constantin Caruntu, Daniel Boda

**Affiliations:** ^1^Department of Dermatology and Allergology, Elias Emergency University Hospital, 011461 Bucharest, Romania; ^2^Department of Plastic and Reconstructive Surgery, “Bagdasar Arseni” Clinical Emergency Hospital, 041915 Bucharest, Romania; ^3^Department of Physiology, “Carol Davila” University of Medicine and Pharmacy, 050474 Bucharest, Romania; ^4^Department of Dermatology, “Prof. N. C. Paulescu” National Institute of Diabetes, Nutrition and Metabolic Diseases, 020475 Bucharest, Romania; ^5^Dermatology Research Laboratory, “Carol Davila” University of Medicine and Pharmacy, 050474 Bucharest, Romania; ^6^Department of Dermatology, Carol Medical Center, 020915 Bucharest, Romania

## Abstract

Cutaneous T-cell lymphoma (CTCL) is the most frequently encountered type of skin lymphoma in humans. CTCL encompasses multiple variants, but the most common types are mycosis fungoides (MF) and Sezary syndrome (SS). While most cases of MF run a mild course over a period of many years, other subtypes of CTCL are very aggressive. The rapidly expanding fields of proteomics and genomics have not only helped increase knowledge concerning the carcinogenesis and tumor biology of CTCL but also led to the discovery of novel markers for targeted therapy. Although multiple biomarkers linked to CTCL have been known for a relatively long time (e.g., CD25, CD45, CD45RA, and CD45R0), compared to other cancers (lymphoma, melanoma, colon carcinoma, head and neck cancer, renal cancer, and cutaneous B-cell lymphoma), information about the antigenicity of CTCL remains relatively limited and no dependable protein marker for CTCL has been discovered. Considering the aggressive nature of some types of CTCL, it is necessary to identify circulating molecules that can help in the early diagnosis, differentiation from inflammatory skin diseases (psoriasis, nummular eczema), and aid in predicting the prognosis and evolution of this pathology. This review aims to bring together some of the information concerning protein markers linked to CTCL, in an effort to further the understanding of the convolute processes involved in this complex pathology.

## 1. Introduction

Early stages of cutaneous T-cell lymphoma (CTCL) are frequently diagnosed as an indolent disease, usually with a long course of evolution. This type of primary lymphoma of the skin is the most frequent seen in humans. However, despite the evolution of medicine and its therapies, the specific cure is not easily found in some cases (15–20%) that have a high relapse rate [1].

The most frequent types of CTCL are mycosis fungoides (MF) and Sezary syndrome (SS). This disease is very complex, with a yet unknown pathogenesis. The development of the disease appears to be tightly connected with the variety of cytokines/chemokines [2]. Most cases of MF evolve over many years, with very slow progression. Early lesions of CTCL typically present as limited skin patches or plaques, called mycosis fungoides (MF), which can progress to tumor stage. In the tumor stage, the process may also involve extracutaneous sites, foremost lymph nodes, and, less frequently, bone marrow and visceral sites [3]. Somewhat different from MF, SS runs a much faster evolving clinical course, with malignant T cells present in the peripheral blood (PB). Patients usually present with lymphadenopathy, erythroderma, fever, and leukemic involvement [4].

CTCL is a type of skin cancer, which represents a significant percentage of all malignancies; therefore early diagnosis and targeted therapy represent the main direction of modern medicine [5]. CTCL, like other dermatological and oncological pathologies, has an important impact on the quality of life of the patient and his family, which is why understanding how CTCL develops may be useful in identifying methods of prevention and perfecting new therapeutic strategies [6, 7].

There are numerous studies that highlight the importance of proteomics as a tool for identifying, through noninvasive/minimally invasive procedures, biomarkers that may allow a more focused approach and management of patient [8–10]. In recent years, the medical community has given a great importance to proteomics, which presents numerous advantages such as the fact that, through it, we can identify molecular changes that sometimes may occur even before any other clinical or laboratory change commonly used for diagnostic; the biomarkers isolated can be used to establish early diagnosis, as well as monitoring and customizing therapy [10–14].

## 2. Cutaneous T-Cell Lymphoma (CTCL)

The increasing number of studies in proteomics and genomics has not only led to a better understanding of the carcinogenesis and tumor biology of CTCL but also led to the discovery of novel markers for targeted therapy. Some tailored target therapies for CTCL are chiefly based on the blockade/inhibition of certain receptors/proteins (IL-2R/CD25, CD4, CD30, and CD52), whose expression by cancer cells can be identified by techniques as immunohistochemistry [15, 16].

Well known diagnostic markers for CTCL include CD2, CD3, CD4, CD5, CD7, CD8, CD14, CD16/56, CD19, CD25, CD45, CD45RA, and CD45R0 [2]; although dissonant with cutaneous B-cell lymphomas and plasma cell disorders, no dependable protein marker for CTCL has been discovered.

Various molecules have been investigated as markers for CTCL, ranging from those involved in general cellular signaling processes, regulation of cellular proliferation, and apoptosis (Jun, Myc, c-myb, p53, STATs, bcl-2, Fas/CD95, and SOCS-3) to those contributing to disease immunopathology like the inhibitory MHC receptors (ILT2/CD85j), NK receptors (p140/KIR3DL2), and dendritic cell defects (CD40). As shown for other tumors, abnormal expression of these molecules influences disease prognosis [17].

It was noted that patients with CTCL had higher serum concentrations of sIL-2R, which can be correlated with lymph node size, large-cell transformation in the skin, or lymph node with increased severity. Regarding the large-cell transformation, it is important to mention that this process is responsible for the increasing concentration of sIL-2R in serum, considering that sIL-2R is produced in low quantities by tissue-based lymphoma cells [18]. Along with sIL-2R, it was demonstrated that patients with Sezary syndrome have elevated levels of *β*_2_-microglobulin (*β*_2_-MG) and neopterin, but only the latter may play a role in determining the prognosis in patients with nonleukemic CTCL [19]. However, studies have shown that these biomarkers have a low specificity for CTCL, as they may be found in elevated concentrations in other malignancies [20].

In a treatment response study [21] differentially expressed genes were significantly associated with treatment-responsive CTCL when compared to treatment-resistant disease. In patients with poor treatment response downregulated genes were involved in epidermal development, Wnt signaling pathway and frizzled signaling, and extracellular matrix pathway, while upregulated genes were those involved in immune response, T-cell activation, mitosis, and apoptosis [21].

Regulatory T cells (Tregs) have two subsets: “natural” Tregs (CD4+CD25+ T cells) which are selected in the thymus and “induced” Tregs which appear in periphery from CD4+ T cells. Although these two categories are very different in many aspects, it is important to mention that they both play a part in tumor immunity [22]. Studies have shown that a decrease in CD4+CD25+ T cells levels is correlated with a high immune response to self- and non-self-antigens [23]. In addition, the most important phenotypic features of this specialized helper T cell are the presence of CD25 component, *α*-chain of the IL-2 (IL-2R*α*), and the transcription factor FoxP3 [24]. It was noted that increasing the levels of CD4+CD25+ T cells can prevent the development of autoimmune conditions, while a decrease in CD25+ T cells or loss of expression of FoxP3 in Tregs may induce a large number of autoimmune diseases [25, 26]. CD25 component is considered to be a stable one in “natural” Tregs and a transient one in “induced” Tregs, but it is important to mention that all Tregs express this marker. Once the exact mechanisms of Treg induction and differentiation are better understood treatment options can be improved [27].

Lymphoma, melanoma, colon carcinoma, head and neck cancer, and renal cancer have all benefited from ample studies on serologically defined antigens. Meanwhile, information about the antigenicity of CTCL remains relatively limited. Forgber et al. tested the sera of 87 cutaneous lymphoma patients and found 64 cases of serum reactivity against lymphoma cells, but the responses were relatively weak, restricted to a few antigens in each case, and heterogeneous. Fourteen different antigens were identified of which only one, vimentin, had been reported before [28].

Recently discovered by Krejsgaard et al. and confirmed in a meta-analysis study by Zhang et al. in 2012 [29], most malignant T cells exhibit ectopic expression of the B-lymphoid tyrosine kinase (Blk), a member of the Src kinase family. Moreover, gene knockdown experiments revealed that Blk promoted the proliferation of malignant T cells in CTCL patients [30] while another study suggested that murine Blk also has tumor-suppressive functions depending on the specific cellular context [29]. Peterson et al. [31] in a recent study of Blk in CTCL provided evidence that the active form of human Blk is able and sufficient to confer cytokine independence to cytokine-dependent lymphoid cells while promoting tumor growth in vivo and supporting growth of lymphoid cells in vitro.

Another recent study analyzed the importance of sCD26 (serum CD26) and TARC (thymus and activation-regulated chemokine) levels in the diagnosis of CTLC. CD26 is a type II transmembrane glycoprotein with enzyme activity, expressed on different tissues such as epithelial cells, endothelial cells, natural killer cells, and a subset of T cells, which selectively removes the N-terminal dipeptide from peptides with proline or alanine in the penultimate position. The soluble form, sCD26, is thought to have the same functions as the membrane form. The sCD26 serum levels are significantly lower in patients with CTLC and psoriasis, while TARC serum levels are significantly higher in patients with atopic dermatitis and CTLC. The authors have shown that these two markers, combined, are helpful in the diagnosis of CTLC [32]. Pierson et al. demonstrated that a decreased CD26 expression is a useful diagnostic marker of peripheral blood involvement in SS and MF patients, but they have also emphasized that reactive T cells in tissue and body fluid specimens often show low levels of CD26 expression; thus this marker should be used in combination with others, in order to facilitate the diagnosis process [33].

Biopsies obtained from CTCL pointed out that the angiogenesis increases with the tumor stage and could play an important part in the pathophysiology and the progression of CTCL. Besides many other angiogenetic and angiostatic factors, VEGF is thought to play a central role in the tumor-associated angiogenesis, its expression being detected even in the early stages. Krejsgaard et al. have demonstrated that advanced stages of CTCL correlate with an increased expression of VEGF and with aberrant activation of its promoting pathways, JAK3 and JNKs. Thus, novel therapies based on the inhibition of these pathways or on the neutralization of VEGF may have an important role in the future and further studies should be conducted in this direction [34].

## 3. Mycosis Fungoides

Almost half of all primary cutaneous lymphomas, as classified by the WHO-EORTC, are represented by mycosis fungoides (MF) [2]. Mycosis fungoides displays a polymorphous clinical picture, varying from patch, plaque, to tumor-stage disease and rarely associates visceral involvement. Clinically discernable lymph node involvement occurs in about 20% and 50% of patients with plaque and tumor-stage MF, respectively [35], while circulating neoplastic cells can be detected even in patients presenting with limited disease [36, 37]. Unfortunately, early stage MF can prove particularly difficult to diagnose mainly due to its benign clinical aspect [38].

Cowen et al. [39] analyzed the possibility of using serum proteomics to distinguish between patients with tumor-stage (T3) mycosis fungoides, patients with psoriasis, and healthy patients. In their study, serum protein profiling successfully distinguished between MF, psoriasis, and healthy controls with acceptable accuracy using the SELDI-TOF Ciphergen MS-based data. Mycosis fungoides detection sensitivity in MF versus controls and MF versus psoriasis groups was 71.4% and 78.6%, respectively, while the specificity kept above 90% in both models. The authors convey the possibility of a unique signature of tumor-stage MF and that perhaps earlier stages of MF would not be as easy to distinguish from other nonneoplastic inflammatory diseases [39]. Moshkovskii et al. [40] attempted to estimate the probability of a correct MF diagnosis based on serum protein profiling using SELDI-TOF MS. The authors found that using their data, differentiation of MF from psoriasis had only 75% specificity and 65.2% sensitivity, an indication of low diagnostic value. However, when comparing protein peaks from MF versus normal controls they found a specificity of 77.7% and a sensitivity of 78.2% which, according to expert scale of AUC (area under curve), is considered to be good [41, 42]. The relatively low values found in MF versus psoriasis patients could be explained by the capacity of direct MS profiling to detect only major proteins, their modifications, and alterations in their serum levels [40].

Manganese superoxide dismutase (SOD2) is an enzyme which is encoded by the SOD2 gene on chromosome 6 and is a member of the superoxide dismutase (SOD) family, whose function is to transform toxic superoxide anion into hydrogen peroxide [43, 44]. Neoplastic cells have an anaerobic metabolism which puts them under intrinsic oxidative stress; due to this process, malignant cells rely on antioxidant enzymes aiming to eliminate reactive oxygen species, which may explain why in malignant lymphocytes in MF there is an overexpression of SOD2 (in epidermal keratinocytes as well as in dermal keratinocytes) [45]. Even though in a mouse model of T-cell lymphoma, SOD2 has a tumor suppressor effect, it was demonstrated that in HIV-infected HUT-78 cells, overexpression of SOD2 may have a tumor-supportive function due to the fact that it increases resistance to heat and radiation [46, 47].

S100A8 is found in the cytoplasm of neutrophils, macrophages, and endothelial cells as calprotectin (S100A8/A9) [48]. Lately it has been documented as a biomarker that is overexpressed in many types of cancers including MF lesions, where it is limited to the epidermis [45]. Even though the cause and role of its overexpression in MF remain unknown, S100A8 appears to be influenced by the hyperproliferative state in psoriasis; this process suggests a similar molecular mechanism in MF [49].

The 15-kDa cytosolic protein FABP5 is a member of the fatty acid binding protein family, involved in the uptake, transport and metabolism of fatty acids, and cellular signaling influencing differentiation and the regulation of cellular growth [50]. The overexpression of FABP5 in the lesional skin of MF patients could be linked to its role in the transport and metabolism of fatty acids in the epidermis, and in turn, the altered lipid metabolism may affect the proliferation and differentiation of keratinocytes [45].

Regarding the differential diagnosis, CD26 soluble serum levels and the expressions of TOX, Tplastin, TWIST, CD 158, and nkP46 have been taken in consideration. TOX is a small DNA binding protein which is regulated in the thymus during positive selection. It has an important role in the CD4+ T-cell development, but after that it is no longer expressed. Recently, some reports have shown that in MF, TOX is overexpressed in mature CD4+ lymphocytes. Furthermore, TOX is a direct target of miR-223, which is considerably reduced in MF/CTCL, leading to an important expression of TOX. Other targets of miR-223 are E2F1 and MEF2C. miRNA are small noncoding RNAs that target mRNA, reducing its translation [51]. Morimura et al. have documented by immunohistochemical staining the TOX expression in the cutaneous lymphocytes from the T-cell infiltrated skin. According to their report, the TOX expression was limited to CD4+ in MF and in CD30+ cells in Lymphomatoid Papulosis (LyP). There was a great amount of TOX expressing cells among infiltrating lymphocytes in biopsies from SS, MF, LyP, primary cutaneous anaplastic large cell lymphoma (PCALCL), adult T-cell lymphoma/leukemia (ATLC), peripheral T-cell lymphoma, and not otherwise specified (PTCL, NOS). Massive infiltration of TOX cells was found in plaques MF, tumor MF, SS, LyP, and PCALC (>30% of infiltrating lymphocytes). A high specific nuclear staining of TOX was found in MF, SS, PTCL, and NOS. On the other hand, the tumor cells, in LyP, PCALCL, and ATLC, presented a weak nuclear staining of TOX. Moreover, the TOX expression was limited to CD4+ cells in MF, while the large anaplastic cells in LyP were positive for CD30+ and CD4+ [52].

Psoriasis, diabetic retinopathy, cardiovascular diseases, rheumatoid arthritis, and cancer are just a few of many diseases that have something in common: pathologic angiogenesis. According to some studies, this pathologic formation of blood vessels can be correlated with different forms of CTCL [53]. Through lymph node biopsies, increased capillary proliferation can be discovered in high-grade non-Hodgkin's lymphoma. The number of blood vessels did not correlate with the grade of the tumor in patients with small cell lymphocytic lymphoma. The progression of CTCL in MF was highlighted by the increased synthesis and expression of matrix metalloproteinases 2 and 9 [54].

Profiling of transcription factors in MF and SS patients has pointed out constitutively active NF-kB, STAT, and their respective signaling pathways as potential markers for target therapy. Several prototypic inhibitors of these targets and altered pathway components have also been identified [55].

**Figure 1:**
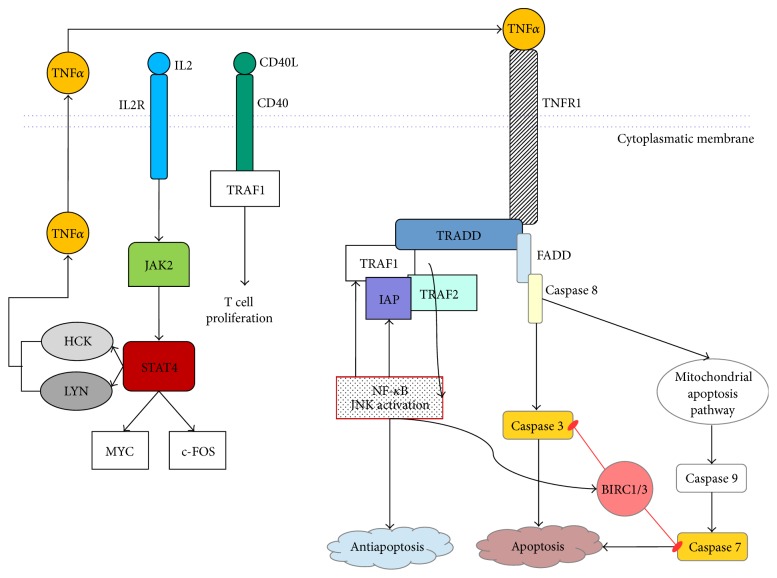
TNF signaling pathway anomalies through antiapoptotic signaling by TNFR1 helped by upregulation of TRAF1 and inhibition of proapoptotic TNFR1 signaling by BIRC1/3 caspase inactivation. Overexpression of CD40L could induce T-cell proliferation through CD40 receptor and TRAF1. IL2R overexpression leads to Jak2 and STAT4 activation which in turn induces oncogene c-MYC, LYN, and HCK expression. Meanwhile LYN and HCK take part in the feedback loop of antiapoptotic TNF signaling by producing endogenous TNF and thus stimulating TNFR1/2 antiapoptotic pathways.

Tracey et al. [56] have shown that there are abnormalities in the TNF signaling pathway in MF tumorigenesis (see Figure 1). Through a cDNA microarray-based approach, the authors [56] identified a total of 27 differentially expressed genes between MF and inflammatory dermatoses (20 upregulated and 7 downregulated in MF cases), including tumor necrosis factor receptor (TNFR) and other oncogenes and apoptosis inhibitors. They designed a 6-gene “signature” (FJX1, STAT4, SYNE1, TRAF1, BIRC3, and Hs.127160) prediction model that may help to differentiate MF from inflammatory dermatoses. The model correctly identified 97% of cases in a blind test performed on 24 MF patients with low clinical stages [56]. cDNA microarray and quantitative RT-PCR expression analyses of peripheral blood samples, using a panel of genes (including STAT4, GATA-3, PLS3, CD1D, and TRAIL), have been shown to identify CTCL patients (who have at least 5% circulating tumor cells) with an overall accuracy of 90% [57, 58].

## 4. Sezary Syndrome

The metastatic potential of tumor cells is dependent on angiogenesis, which creates the conditions for tumor growth and progression [59, 60]. Angiopoietins represent a family of ligands for endothelium-specific tyrosine kinase Tie2 receptor. This family of proteins consists of 4 structurally similar members: Ang-1, Ang-2, Ang-3, and Ang-4 of which Ang-1 and Ang-2 have been identified to have angiogenetic properties, similar to VEGF [61–63]. Cells from MF skin lesions express Ang-2, but the serum levels of this protein are elevated only in patients with Sezary syndrome (SS), which may indicate that serum Ang-2 is produced by circulating tumor cells in SS [4, 64]. Kawaguchi et al. showed in a study published in 2014 that serum concentration of Ang-2 was increased, even in patients with MF, when the disease progressed, thus demonstrating that Ang-2 could be used as a disease activity marker [65].

TOX staining may also be useful in the differential diagnosis between SS and erythrodermic dermatitis. The first mentioned presented more than 50% nuclear staining of the infiltrating lymphocytes, while the last ones had a slightly dim nuclear staining of TOX (~25%). In addition, c-MYC did not have a significant contribution to the differential diagnosis [66]. Tplastin, a member of the fimbrin/plastin family expressed by epithelial tissues and nonhematopoietic mesenchymal cells, did not show a significantly higher expression in SS than in the benign erythrodermic inflammatory diseases (EID). In contrast, the detection of CD 158K/KIR3DL2 (a killer immunoglobulin-like receptor usually expressed by some circulating T CD8+ lymphocytes, NK cells, and recently some reports suggested that it might be expressed by some subsets of CD4+ T cells) transcripts using RT-PCR was significantly overexpressed in SS, in comparison with EID, and may be used as a diagnosis marker even in the early stages of SS [67].

In another study, Michel et al. have demonstrated for the first time that the combination of five biomarkers (PL53, TWIST, CD158K, KIR3DL2, and NKp46) using PCR has a high importance in the early diagnosis of SS [68]. In advanced MF/SS increased expression of Twist protein (a transcription factor which blocks p53 and inhibits c-MYC induced apoptosis, believed to promote the progression of solid tumors) was found through RT-PCR detection, but further studies are needed in order to correlate the high expression with the stages of these diseases [69]. NK46p belongs to natural cytotoxicity receptor (NCR) families of natural killer (NK) receptors. This receptor is frequently expressed by neoplastic cells in SS and it may be used as a diagnosis marker in the blood and the skin using RT-PCR [70].

## 5. Conclusions

Given the high prevalence of CTCL, it is imperative to determine specific biomarkers in order to distinguish between benign and aggressive prognostic courses. The diagnosis of CTCL requires a more holistic approach through which molecular findings are to be integrated with clinical, histological, and immunophenotypic data. Thus, future studies should be aimed at defining appropriate molecules with high sensitivity and specificity for the evaluation of disease treatment and prognosis. Moreover at-risk patients would benefit from diagnostic markers in order to prevent disease progression and late diagnosis, when appropriate therapies are of little efficiency. Establishing accurate protein markers would also be helpful for identifying target therapies.
